# Distribution of Japanese Encephalitis Virus, Japan and Southeast Asia, 2016–2018

**DOI:** 10.3201/eid2601.190235

**Published:** 2020-01

**Authors:** Ryusei Kuwata, Shun Torii, Hiroshi Shimoda, Supriyono Supriyono, Thanmaporn Phichitraslip, Noppadol Prasertsincharoen, Hitoshi Takemae, Reu Caesar James Taga Bautista, Valeen Drex Bendette Mendio Ebora, Jose Alexander Cabiling Abella, Alan Payot Dargantes, Upik Kesumawati Hadi, Agus Setiyono, Emmanuel Tugbang Baltazar, Luzviminda Tadeja Simborio, Srihadi Agungpriyono, Sathaporn Jittapalapong, Worawut Rerkamnuaychoke, Eiichi Hondo, Ken Maeda

**Affiliations:** Yamaguchi University, Yamaguchi, Japan (R. Kuwata, S. Torii, H. Shimoda, Supriyono, K. Maeda);; Kasetsart University, Bangkok, Thailand (T. Phichitraslip, N. Prasertsincharoen, S. Jittapalapong);; Nagoya University, Nagoya, Japan (H. Takemae, E. Hondo);; Central Mindanao University, Musuan, the Philippines (R.C.J.T. Bautista, V.D.B.M. Ebora, J.A.C. Abella, A.P. Dargantes, E.T. Baltazar, L.T. Simborio);; Bogor Agricultural University, Bogor, Indonesia (U.K. Hadi, A. Setiyono, S. Agungpriyono);; Rajamangala University of Technology, Chonburi, Thailand (W. Rerkamnuaychoke)

**Keywords:** Japanese encephalitis virus, *Culex tritaeniorhynchus*, pig, wild boar, vector-borne infections, mosquitoborne disease, Japan, Thailand, the Philippines, Indonesia, meningitis/encephalitis, Southeast Asia, epidemiology, arbovirus, arthropodborne virus, viruses, surveillance, GIII, GIV, zoonoses

## Abstract

During 2016–2018, we conducted surveillance for Japanese encephalitis virus (JEV) in mosquitoes and pigs in Japan, Thailand, the Philippines, and Indonesia. Phylogenetic analyses demonstrated that our isolates (genotypes Ia, Ib, III, IV) were related to JEV isolates obtained from the same regions many years ago. Indigenous JEV strains persist in Asia.

The locations of epidemics of arthropodborne viruses (arboviruses) are strongly associated with the distribution of their vectors. In general, the distribution of arboviruses can expand through the dispersal, transfer, and migration of their vector arthropods and reservoir animals. Mosquitoes transmit a variety of viral pathogens (e.g., dengue, Zika, and chikungunya viruses) and have caused a number of arboviral epidemics throughout the world (*1*). Japanese encephalitis virus (JEV; family *Flaviviridae*, genus *Flavivirus*) is a mosquitoborne arbovirus that causes a severe form of encephalitis in humans. JEV is distributed across most of Asia, the western Pacific, and northern Australia (*2*). The World Health Organization has estimated that the annual number of Japanese encephalitis cases worldwide exceeds 60,000 (*2*). JEV is transmitted primarily by mosquitoes of the *Culex vishnui* subgroup, principally *Cx*. *tritaeniorhynchus* Giles; pigs and wading ardeid birds, such as egrets and herons, are known to be the major amplifying hosts (*3*).

On the basis of their genome sequences, JEVs are classified into 5 genotypes (*4*). JEV genotype I (GI), which has been further classified into subgenotypes GIa and GIb, and JEV GIII are the dominant lineages and have been detected widely throughout Asia. JEV GII is the thirdmost common lineage and has been found in Indonesia, Singapore, South Korea, Malaysia, and Australia. JEV GIV and GV are rare lineages; only a few viruses of these genotypes have been isolated from Indonesia, Malaysia, and China as of October 2019. Over the past 30 years, JEV GIa has displaced GIII as the dominant lineage in many countries of Asia (*5*). Although the origin and spreading pattern of JEV genotypes across the world have been investigated in some reports (*6*,*7*), the exact mechanisms of JEV genotype shift remain unclear.

## The Study

To study the epidemiology of arbovirus infection, we, an international team of researchers in Japan, Thailand, the Philippines, and Indonesia, conducted arbovirus surveillance in our respective countries during 2016–2018 with the support of our governments. In each country, we collected mosquitoes in and around cattle or pig housing using sweeping nets and aspirators. We collected mosquitoes that had digested blood in their midguts. We identified their species and sorted them into pools, which we used for virus isolation. We also collected serum samples from pigs and wild boars from each country to use for virus isolation.

We passed homogenized mosquito or serum samples through 0.45-μm filters (Corning Inc., https://www.corning.com) and inoculated filtrates onto monolayers of 3 culture cell lines (mammalian cell lines Vero9013 and BHK-21 and mosquito cell line C6/36). We assessed cytopathic effect (CPE) daily and collected supernatants from cells that exhibited CPE. If we observed no CPE, we passaged the cells 5 times for 7 days each, after which, if virus was present, CPE should have become apparent. We extracted RNA from culture supernatants using the QIAamp Viral RNA Mini Kit (QIAGEN, https://www.qiagen.com) and subjected the resulting RNA to reverse transcription PCR (RT-PCR) using the QIAGEN One-Step RT-PCR Kit and 2 universal flavivirus-specific primer sets (MAMD and cFD2 or FU2 and cFD3) (*8*,*9*) to screen for flaviviruses. To determine genome sequences, we used the QIAGEN One-Step RT-PCR Kit, TaKaRa LA RT-PCR Kit version 2.1 (Takara Bio, https://www.takarabio.com), and Invitrogen 5′ RACE System for Rapid Amplification of cDNA Ends version 2.0 (https://www.thermofisher.com) as needed in combination with several JEV-specific primers (Appendix Table 1).

Of 945 pig serum samples, we selected 56 candidate samples for virus isolation on the basis of their RT-PCR results with the MAMD and cFD2 primers, and from these samples, we obtained the full or partial genome sequences of 5 JEV isolates (Appendix Table 2). Out of a total of 22,277 mosquitoes comprising >16 species, we obtained the full or partial genome sequences of only 2 JEV isolates (Appendix Table 3). Overall, we obtained the full-genome sequence of 4 of the 7 JEV isolates and the partial genome sequence (envelope gene) of the remaining 3 isolates (DDBJ accession nos. LC461956–62; Table).

**Table:** JEV isolates, Japan and Southeast Asia, 2016–2018*

Virus isolate	Country	Province or city, island	Source	Collection date	DDBJ accession no.
JEV/MQ/Yamaguchi/803/2016	Japan	Yamaguchi	*Culex tritaeniorhynchus* mosquito	2016 Sep 8	LC461956†
JEV/MQ/Yamaguchi/804/2016	Japan	Yamaguchi	*C. tritaeniorhynchus* mosquito	2016 Sep 8	LC461957‡
JEV/sw/Thailand/185/2017	Thailand	Nakornnayok	Pig serum sample	2017 Aug 14	LC461958‡
JEV/sw/Mindanao/K3/2018	The Philippines	Butuan, Mindanao	Pig serum sample	2018 Mar 9	LC461959†
JEV/sw/Mindanao/K4/2018	The Philippines	Butuan, Mindanao	Pig serum sample	2018 Mar 9	LC461960‡
JEV/sw/Bali/93/2017	Indonesia	Denpasar, Bali	Pig serum sample	2017 Oct 17	LC461961‡
JEV/sw/Bali/94/2017	Indonesia	Denpasar, Bali	Pig serum sample	2017 Oct 17	LC461962†

*JEV, Japanese encephalitis virus. †Envelope gene sequence. ‡Full-genome sequence.

A preliminary study we performed showed that many pigs in these countries possessed antibodies against JEV (K. Maeda, unpub. data). We found that JEV was more often isolated from serum samples from JEV antibody–negative pigs in farms where JEV seroprevalence was low. Because JEV isolation seems to be difficult in endemic regions, we suggest selecting younger pigs, which are less likely to be JEV antibody positive, for virus isolation studies to increase the chances of success.

The 2 JEV isolates we recovered in Japan, JEV/MQ/Yamaguchi/803/2016 and JEV/MQ/Yamaguchi/804/2016, were GIa and not GIII. In a phylogenetic analysis, these viruses clustered with JEV isolates recovered from Japan (in 2013 and 2009), China (in 2007), and South Korea (in 2010) (Figure, panel A). The 2 viruses collected in Japan in 2016 were most closely related to viruses recovered in 2013 from the same site that had been sampled in our previous study (*10*), suggesting that this strain has been maintained in the Yamaguchi area of Japan since at least 2013.

**Figure:**
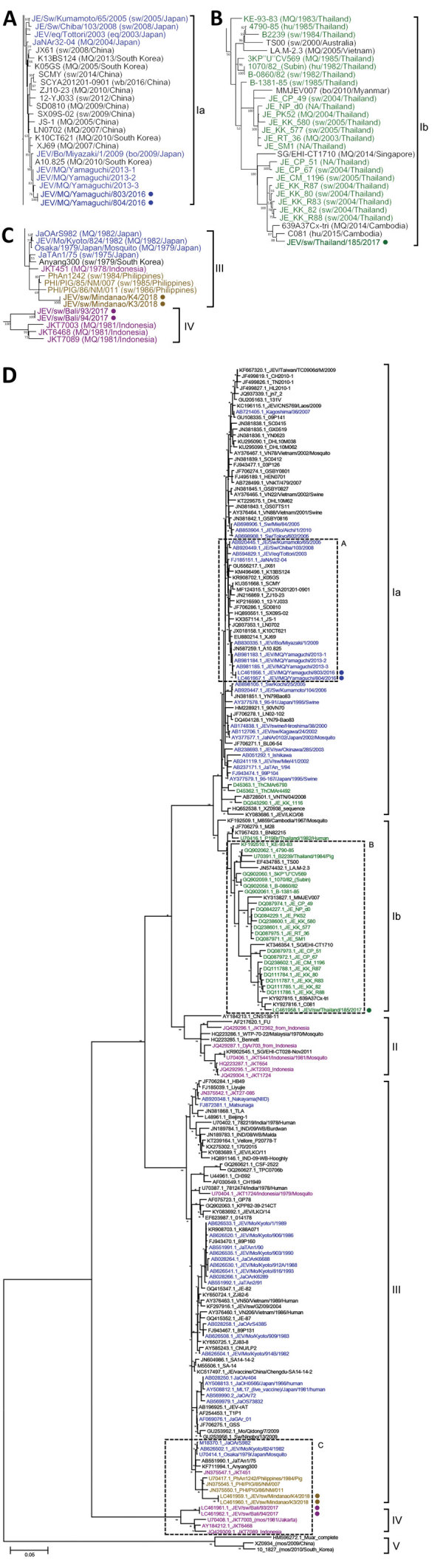
Maximum-likelihood phylogeny of JEV isolates, Japan and Southeast Asia, 2016–2018 (circles), and reference isolates constructed on the basis of the envelope gene sequence (1,500 nt). A) Genotype Ia (GIa); B) genotype GIb; C) genotypes GIII and GIV; D) parent tree showing all genotypes. Tree was reconstructed by using MEGA6 (https://megasoftware.net) with 100 bootstraps under the general time-reversible model. JEVs isolated in Japan (blue), Thailand (green), the Philippines (yellow), and Indonesia (maroon) are indicated. In panels A–C, origin, year, and country of isolation are provided in parentheses; in panel D, DDBJ and GenBank accession numbers are provided. bo, bovine; eq, equine; hu, human; JEV, Japanese encephalitis virus; MQ, mosquito; NA, data not available; sw, swine; wb, wild boar.

The JEV isolate we obtained from Thailand, JEV/sw/Thailand/185/2017, was GIb and clustered with other JEV isolates detected in Thailand during 1985–2005 (*11*), as well as isolates from Myanmar in 2010, Cambodia in 2014 and 2015, and Singapore in 2014 (Figure, panel B). Thus, these JEVs have been maintained in Thailand and other parts of the southern peninsula of continental Asia for >30 years.

JEV is distributed extensively throughout the Philippines (*12*). However, only 3 JEV GIII isolates from the Philippines (which were obtained from pigs during 1984–1986) were available for genetic analysis. Our 2 Philippines-derived JEV isolates, JEV/sw/Mindanao/K3/2018 and JEV/sw/Mindanao/K4/2018, clustered with these isolates (Figure, panel C). Our 2 Indonesia JEV isolates, JEV/sw/Bali/93/2017 and JEV/sw/Bali/94/2017, were obtained from the island of Bali, where a JEV vaccination program began in March 2018 (*13*). These JEV isolates clustered with other Indonesia isolates obtained in the 1980s (Figure, panel C) (*14*).

## Conclusions

In summary, our phylogenetic analysis revealed that the JEV isolates we obtained from Japan were GIa, the isolate from Thailand was GIb, the isolates from the Philippines were GIII, and the isolates from Indonesia were GIV (Figure, panels A–D; Appendix Figure). These results indicated that JEV GIII and GIV are still active and being maintained in parts of Asia.

Our data demonstrate that a number of the JEV isolates we obtained in select countries of Southeast Asia during 2016–2018 were phylogenetically related to isolates reported in the same country in the 1980s, suggesting that some JEV strains have been maintained in their corresponding regions. Contrary to our expectation, the JEV transmission cycle seems to have been maintained indigenously. JEV strains are presumed to be transferred between JEV-endemic regions by movement of arthropod vectors and bird reservoirs. Nonetheless, we infer that fixation of an invading JEV strain into a new region is difficult unless the new strain possesses properties advantageous for virus growth and expansion (*15*). However, the genotype shift from GIII to GIa has occurred in East Asia since the 1990s, indicating that GIa must have had some sort of growth advantage over GIII that permitted its spreading to and expansion in these countries. Our findings that JEV strain invasion in Asia is infrequent could assist in public health decision-making regarding vaccine formulation and campaign strategies.

Appendix More information about the distribution of Japanese encephalitis virus, Japan and Southeast Asia, 2016–2018.
