# Supplementary material for: Distribution of Japanese Encephalitis Virus, Japan and Southeast Asia, 2016–2018
Source: Emerg Infect Dis. 2020 Jan;26(1):125–8. doi: 10.3201/eid2601.190235 (PMC6924880; doi:10.3201/eid2601.190235)
Supplement: Appendix — More information about the distribution of Japanese encephalitis virus, Japan and Southeast Asia, 2016–2018. [file 19-0235-Techapp-s1.pdf]

Article DOI: https://doi.org/10.3201/eid2601.190235

# Distribution of Japanese Encephalitis Virus, Japan and Southeast Asia, 2016–2018

## Appendix

**Appendix Table 1.** List of primers for JEV*

| Primer name | Sequence | Sense/Antisense |
|---|---|---|
| JEV136F | 5′- ATCAATATGCTGAAACGCGG –3′ | Sense |
| JEV197R | 5′- ACTACCCTCTTCACTCCCAC –3′ | Antisense |
| JEV541F | 5′- GTCATCGTGATCCCCACCTC –3′ | Sense |
| JEV898F | 5′- CTTGGMTGGATGCTTGGCAG –3′ | Sense |
| JEV922R | 5′- TGTTGCTGCCAAGCATCCA –3′ | Antisense |
| JEV980R | 5′- AAACTGTATGCCGGAGCGACC –3′ | Antisense |
| JEV1306F | 5′- AAAGGAAGCATTGACACATG –3′ | Sense |
| JEV1684F | 5′- AGAGAACTTCTCATGGAATTTG –3′ | Sense |
| JEV2335F | 5′- TTTGGTGGAATGTCCTGGAT –3′ | Sense |
| JEV3135R | 5′- CTCTCCGAAGACDGCCCTCTCAAG –3′ | Antisense |
| JEV3635F | 5′- TTCCTGCGGTTTTGGGGGCCCT –3′ | Sense |
| JEV3689R | 5′- GTGTAAGTGATGCCCCCAAGCATCAG –3′ | Antisense |
| JEV4330F | 5′- ATGGCAGTGTCTTACGTGGT –3′ | Sense |
| JEV5035F | 5′- AATGGAGACATCATAGGC –3′ | Sense |
| JEV5026F | 5′- CTGGACTCCAATGGAGACAT –3′ | Sense |
| JEV5950F | 5′- TCTCCCATAACCAGTGCAAG –3′ | Sense |
| JEV6113R | 5′- GGCATGTGTATGTTGTCTA –3′ | Antisense |
| JEV7115R | 5′- GATGTGGTCACGTATTC –3′ | Antisense |
| JEV7381F | 5′- ACTGATGTGCCYGAACTGGAAAG –3′ | Sense |
| JEV7670R | 5′- AAGGAGGGCTTGTCAGCYTTCTT –3′ | Antisense |
| JEV7930F | 5′- GGAGGCTGGAGCTACTACGC –3′ | Sense |
| JEV8689F | 5′- GCCAATGTCACCACAATGGC –3′ | Sense |
| JEV9454F | 5′- ATGGACGTGATATCAAGAGAAGA –3′ | Sense |
| JEV10490R | 5′- GTCTACCCAGTATCCGGTGG –3′ | Antisense |
| JEV10527F | 5′- GACTGGGTTAACAAATCTGACAA –3′ | Sense |

| Primer name | Sequence | Sense/Antisense |
|---|---|---|
| JEV10564F | 5′- CCTCAGAACCGTCTCGGAAG –3′ | Sense |
| JEV10970R | 5′- AGATCCTGTGTTCTTCCTCACCA –3′ | Antisense |
| MAMD† | 5′- AACATGATGGGRAARAGRGARAA –3′ | Sense |
| cFD2‡ | 5′- GTGTCCCAGCCGGCGGTGTCATCAGC –3′ | Antisense |

*JEV, Japanese encephalitis virus.

†Obtained from Scaramozzino et al. (*1*).

‡Obtained from Kuno et al. (*2*).

**Appendix Table 2.** Information on pig sera used for the isolation of JEV in this study*

| | | No. of tested | | Isolates |
|---|---|---|---|---|
| Site of sample collection | Month of sample collection | RT-PCR | Isolation | of JEV |
| Thailand | | | | |
| Nakorn Nayok | May-August 2017 | 100 | 20 | 1 |
| Prachinburi | July 2017 | 100 | – | – |
| Sakon Nakhon | August 2017 | 51 | 12 | 0 |
| Lop Buri | August 2017 | 40 | – | – |
| Saraburi | August 2017 | 60 | – | – |
| Tak | February 2018 | 256 | 7 | 0 |
| Ratchaburi | February 2018 | 122 | – | – |
| The Philippines | | | | |
| Butuan, Agusan del Norte | March 2018 | 56 | 12 | 2 |
| Santo Tomas, Davao del Norte | March 2018 | 12 | – | – |
| Tagum, Davao del Norte | March 2018 | 6 | – | – |
| Digos, Davao del Sur | March 2018 | 11 | – | – |
| Indonesia | | | | |
| Bengkulu, Sumatera (wild boar) | May 2017 | 26 | – | – |
| Denpasar, Bali | October 2017 | 105 | 5 | 2 |
| Total | | 945 | 56 | 5 |

*JEV, Japanese encephalitis virus; RT-PCR, reverse transcription PCR.

**Appendix Table 3.** Information on mosquitoes used for the isolation of Japanese encephalitis virus in this study

| Site (place, month/year) | *Culex tritaeniorhynchus* | *Cx. pseudovishnui* | *Cx. vishnui* | *Cx. quinquefasciatus* | *Cx. pipiens* pallens | *Cx. gelidus* | *Cx. hutchinsoni* | *Cx. fscocephala* | *Cules* sp. | *Anopheles sinensis* | *A. vagus* | *A. campestris* | *Anopheles* sp. | *Aedes albopictus* | *Ae. vexans* | *Ae. japonicus* | *Armigeres sublbatus* | *Mansonia uniformis* |
|---|---|---|---|---|---|---|---|---|---|---|---|---|---|---|---|---|---|---|
| Yamaguchi, Japan (cowshed, 2014 total) | 3457* | | – | – | 29 | – | – | – | – | 770 | – | – | – | 7 | 3 | 15 | 9 | – |
| Yamaguchi, Japan (cowshed, 2015 total) | 1701 | 477 | – | – | 56 | – | – | – | – | 1323 | – | – | – | 33 | 12 | 6 | – | – |
| Yamaguchi, Japan (cowshed, 2016 total) | 7218† | 822 | – | – | 36 | – | – | – | – | 1424 | – | – | – | 33 | 11 | 1 | – | – |
| Khlong Luang, Thailand (cowshed, Nov 2015) | 635 | – | 47 | – | – | 608 | – | – | – | – | – | 2 | 160 | 2 | – | – | 1 | 8 |
| Thanyaburi, Thailand (pigpen, Sep 2016) | 1121 | – | 78 | 59 | – | 1180 | – | – | – | – | 18 | – | – | – | – | – | – | 13 |
| Mindanao, the Philippines (cowshed, Jul 2015) | 63 | – | 13 | – | – | 21 | 3 | 3 | – | – | 5 | – | – | – | – | – | – | – |
| Mindanao, the Philippines (cowshed, Mar 2017) | 176 | – | 63 | – | 61 | 41 | – | 74 | 4 | – | – | – | – | – | – | – | – | – |
| Bogor, Indonesia (cowshed, Oct 2015) | 2 | – | – | 10 | – | – | – | – | – | – | – | – | 3 | – | – | – | 4 | |
| Bogor, Indonesia (cowshed, Jun 2016) | 24 | – | 28 | 1 | – | 22 | 13 | – | 32 | – | 96 | – | 2 | 6 | – | – | 6 | – |
| Bogor, Indonesia (cowshed, Nov 2016) | 73 | – | – | – | – | 19 | 5 | – | – | – | 26 | – | – | – | – | – | 5 | – |
| Total | 14470 | 1299 | 229 | 70 | 182 | 1891 | 21 | 77 | 36 | 3517 | 143 | 2 | 165 | 81 | 26 | 22 | 25 | 21 |

| Site (place, month/year) | *Culex tritaeniorhynchus* | *Cx. pseudovishnui* | *Cx. vishnui* | *Cx. quinquefasciatus* | *Cx. pipiens* pallens | *Cx. gelidus* | *Cx. hutchinsoni* | *Cx. fscocephala* | *Cules* sp. | *Anopheles sinensis* | *A. vagus* | *A. campestris* | *Anopheles* sp. | *Aedes albopictus* | *Ae. vexans* | *Ae. japonicus* | *Armigeres sublbatus* | *Mansonia uniformis* |
|---|---|---|---|---|---|---|---|---|---|---|---|---|---|---|---|---|---|---|

*The number of *Cx. tritaeniorhynchus* and *Cx. pseudovishnui* were mixed.

†Two Japanese encephalitis virus isolates were obtained.

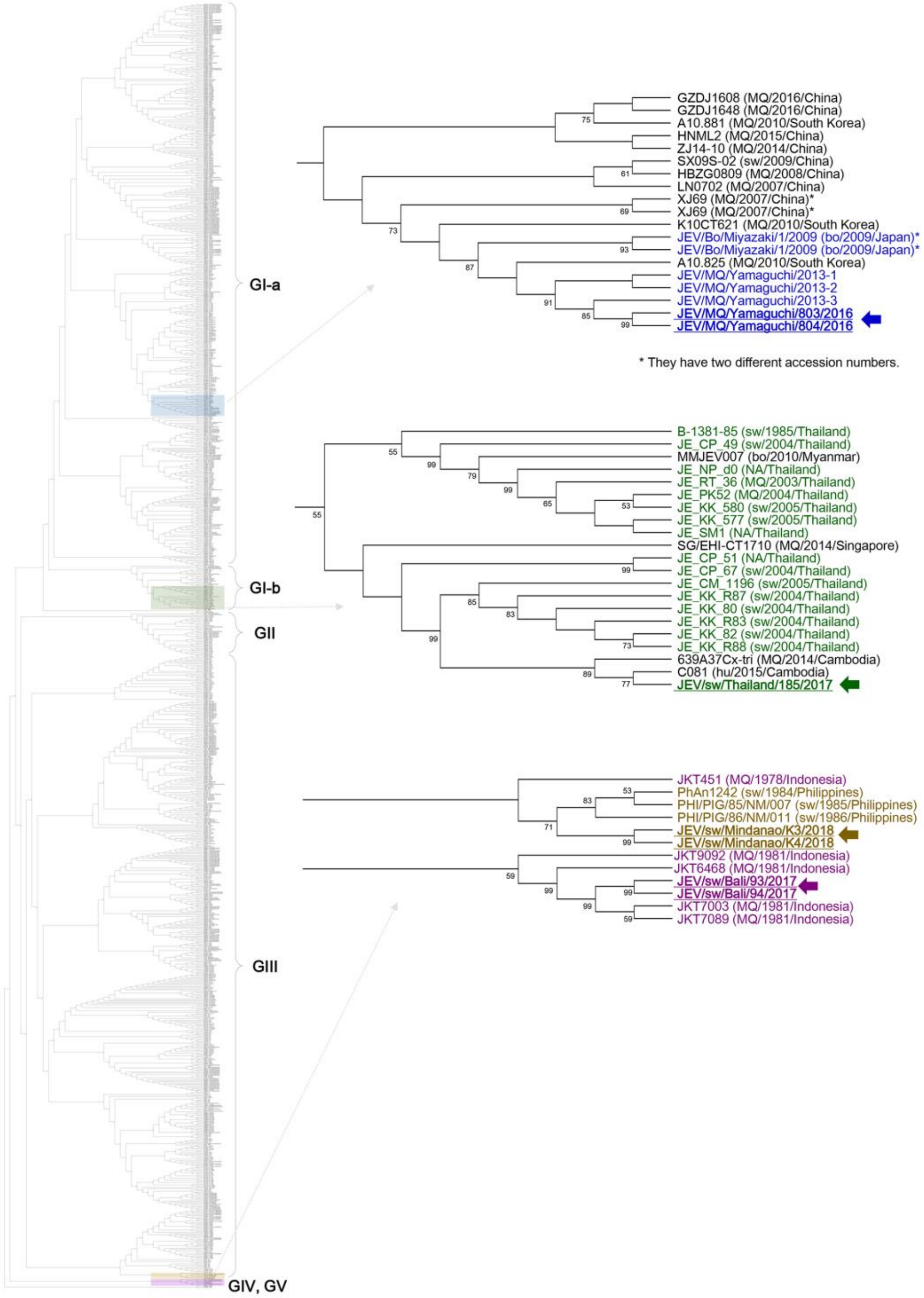


**Appendix Figure.** Maximum-likelihood phylogeny of 1143 strains of Japanese encephalitis virus (JEV) based on the envelope (E) gene sequences (1500 nt from each viral genome). The tree was reconstructed using MEGA6 with 50 bootstraps under the generalized time reversible model. In this tree, blue, green, yellow, and purple indicate the JEV strains that were isolated from Japan, Thailand, the Philippines, and Indonesia, respectively. Arrows indicate the JEV strains obtained in the present study.

This analysis revealed that the new JEVs isolated from Japan, Thailand, the Philippines, and Indonesia belong to GI-a, GI-b, GIII, and GIV, respectively. Origin, year, and country for each JEV strain are provided in parentheses. bo, bovine; eq, equine; hu, human; G, genotype; MQ, mosquito; NA, data not available; sw, swine; wb, wild boar.

### Appendix References


1. Scaramozzino N, Crance JM, Jouan A, DeBriel DA, Stoll F, Garin D. Comparison of flavivirus universal primer pairs and development of a rapid, highly sensitive heminested reverse transcription-PCR assay for detection of flaviviruses targeted to a conserved region of the NS5 gene sequences. J Clin Microbiol. 2001;39:1922–7. PubMed http://dx.doi.org/10.1128/JCM.39.5.1922-1927.2001

2. Kuno G, Chang GJ, Tsuchiya KR, Karabatsos N, Cropp CB. Phylogeny of the genus *Flavivirus.* J Virol. 1998;72:73–83. PubMed
